# Decision making in out-of-hospital cardiac arrest: what should come first?

**DOI:** 10.1007/s12471-024-01856-w

**Published:** 2024-02-07

**Authors:** Arnoud W. J. van ’t Hof, Thijs S. R. Delnoij, Iwan C. C. van der Horst

**Affiliations:** 1 Department of Cardiology, Maastricht University Medical Centre, Maastricht, The Netherlands; 2 Department of Cardiology, Zuyderland Medical Centre, Heerlen, The Netherlands; 3 Cardiovascular Research Institute Maastricht, Maastricht, The Netherlands; 4 Department of Intensive Care, Maastricht University Medical Centre, Maastricht, The Netherlands

In acute and critical care, time is a crucial factor in decision making. Delayed treatment is associated with deterioration in organ function, quality of life and, eventually, life itself. Therefore, healthcare pathways aim to minimise the time from initial medical contact to eventual treatment. To achieve this, symptoms must be apparent, skilled caregivers should make the correct diagnosis, and treatment should be available shortly after diagnosis, as recently described in this journal [1]. However, variability in symptom presentation and diagnostic accuracy introduces uncertainty. Symptoms can differ among different individuals depending on, for example, age and sex. A certain accuracy exists for all diagnostic tests, but this accuracy is seldom a certainty, for example 100%. This is also true in patients with ST-elevation myocardial infarction (STEMI) after out-of-hospital cardiac arrest (OHCA).

In the Netherlands, established networks have significantly improved survival rates of OHCA, with neurological outcomes heavily dependent on prompt action, bystander basic life support and early defibrillation. Rapid primary percutaneous coronary intervention (PCI) is the main driving factor to ensure the best outcome in OHCA patients with STEMI [2, 3]. Furthermore, coronary angiography is valuable, as in these patients an increased incidence of acute coronary occlusion and more complex non-culprit coronary artery disease is observed compared to STEMI patients without cardiac arrest [4].

In this issue of the *Netherlands Heart Journal*, the results of a retrospective study on patients with acute myocardial infarction after OHCA are presented [5]. The researchers focused on the effect of ruling out severe brain haemorrhage before coronary intervention on time, as well as on making a diagnosis that would contradict coronary intervention, including treatment with antithrombotic and antiplatelet therapy. In their study, the effect on time was significant, with a mean delay longer than an hour. However, the number of patients with a visible traumatic head injury on the scan was 7 of 11 (64%). Nevertheless, none of the patients had intracranial haemorrhage. In addition, in only a very low number of cases did a critical diagnosis contradict the treatment. These results complement previous studies. Recently, another multicentre retrospective study from Japan showed that 8 of 345 patients (2.3%) with a CT scan after OHCA had collapse-related traumatic intracranial haemorrhage [6]. In a single-centre prospective study from the United States, all OHCA patients underwent a CT scan. Intracranial haemorrhage was observed in 3 of 85 patients (3.5%) [7]. A higher prevalence of intracranial haemorrhage was also found in a meta-analysis of 54,349 cases of non-traumatic OHCA, in which the incidence was 4.3% [8].

Based on these results, one could reconsider current strategies to optimally treat patients with STEMI after OHCA. This reconsideration could mean that, for the majority of patients, the benefit of early treatment is so high that any delay based on additional diagnostics should be prevented. Another reconsideration might be that, although only a minority benefits, further diagnostics should be included and optimised to avoid serious treatment-related adverse events, such as severe cerebral bleeding. Adding to this consideration is the fact that some conditions can mimic STEMI, such as traumatic cardiac contusion of subarachnoid bleeding.

Both strategies have benefits and harms, and it is up to experts to interpret the available evidence to guide colleagues in optimising care. In the case of STEMI after OHCA with evidence of (minor) head trauma, the inclination may be towards immediate coronary intervention unless the likelihood of severe cerebral haemorrhage or injury is so high that performing immediate PCI is not the primary focus. Examples might be significant head bleeding, severely fractured skull, unresponsive and enlarged, dysmorphic pupils.

Beside STEMI after OHCA with head trauma, other medical emergencies have similar, opposing treatment challenges. Acute heart failure in patients with sepsis requires fluid removal to optimise oxygenation versus resuscitation for hypovolaemia and diminished organ perfusion. Ventricular arrhythmias in patients with poor ventricular function and heart failure might require proarrhythmic inotropes. Severe respiratory failure in patients with right-sided heart failure might require right ventricular afterload, increasing mechanical ventilation. These challenges urge case-specific decisions with little evidence-based guidance. The question arises: should individualised care be prioritised at the expense of potentially delaying optimal treatment for the majority? At the very least, we should inform caregivers and patients that there is no standard method of treatment without risk. We aim for the best for the most, as aiming for the best for each individual is associated with unfavourable delays in most cases. The strategy to treat as soon as possible, except in patients with evident comorbidities or concomitant diseases that contradict this approach, seems optimal, especially in OHCA with STEMI and minor head trauma.
